# Machine Learning–Based Identification of Target Groups for Thrombectomy in Acute Stroke

**DOI:** 10.1007/s12975-022-01040-5

**Published:** 2022-06-07

**Authors:** Fanny Quandt, Fabian Flottmann, Vince I. Madai, Anna Alegiani, Clemens Küpper, Lars Kellert, Adam Hilbert, Dietmar Frey, Thomas Liebig, Jens Fiehler, Mayank Goyal, Jeffrey L. Saver, Christian Gerloff, Götz Thomalla, Steffen Tiedt, the GSR investigators and the VISTA-Endovascular Collaborators, J. Berrouschot, A. Bormann, G. Bohner, C. H. Nolte, E. Siebert, S. Zweynert, F. Dorn, G. C. Petzold, F. Keil, W. Pfeilschifter, G. F. Hamann, M. Braun, B. Eckert, J. Röther, A. Alegiani, J. Fiehler, C. Gerloff, G. Thomalla, C. Kraemer, K. Gröschel, T. Uphaus, L. Kellert, S. Tiedt, C. Trumm, T. Boeckh-Behrens, S. Wunderlich, A. Ludolph, M. Petersen, F. Stögbauer, U. Ernemann, S. Poli, P. Khatri, M. Bendszuz, S. Bracard, J. Broderick, B. Campbell, A. Ciccone, A. Davalos, S. Davis, A. Demchuk, H. C. Diener, D. Dippel, G. A. Donnan, X. Ducrocq, J. Fiehler, D. Fiorella, G. Ford, M. Goyal, W. Hacke, M. Hill, R. Jahan, E. Jauch, T. Jovin, C. Kidwell, K. R. Lees, D. S. Liebeskind, C. B. Majoie, S. Martins, P. Mitchell, J. Mocco, K. Muir, R. G. Nogueira, J. L. Saver, W. J. Schonewille, A. H. Siddiqui, G. Thomalla, T. A. Tomsick, A. S. Turk, W. H. van Zwam, P. White, S. Yoshimura, O. O. Zaidat

**Affiliations:** 1 Department of Neurology, University Medical Center Hamburg-Eppendorf, Hamburg, Germany; 2 Department of Diagnostic and Interventional Neuroradiology, University Medical Center Hamburg-Eppendorf, Hamburg, Germany; 3 Charité Lab for Artificial Intelligence in Medicine-CLAIM, Charité - Universitätsmedizin Berlin, Berlin, Germany; 4 QUEST Center for Transforming Biomedical Research, Berlin Institute of Health, Charité - Universitätsmedizin Berlin, Berlin, Germany; 5 School of Computing and Digital Technology, Faculty of Computing, Engineering and the Built Environment, Birmingham City University, Birmingham, UK; 6 Department of Neurology, University Hospital, LMU Munich, Munich, Germany; 7 Institute of Neuroradiology, University Hospital, LMU Munich, Munich, Germany; 8 Department of Radiology, Foothills Medical Centre, University of Calgary, Calgary, AB Canada; 9 Department of Neurology and Comprehensive Stroke Center, David Geffen School of Medicine, University of California Los Angeles, Los Angeles, CA USA; 10 Institute for Stroke and Dementia Research, University Hospital, LMU Munich, Feodor-Lynen-Straße 17, 81377 Munich, Germany

**Keywords:** Machine learning, Endovascular thrombectomy, Real-world data, Outcome prediction, Stroke

## Abstract

Whether endovascular thrombectomy (EVT) improves functional outcome in patients with large-vessel occlusion (LVO) stroke that do not comply with inclusion criteria of randomized controlled trials (RCTs) but that are considered for EVT in clinical practice is uncertain. We aimed to systematically identify patients with LVO stroke underrepresented in RCTs who might benefit from EVT. Following the premises that (i) patients without reperfusion after EVT represent a non-treated control group and (ii) the level of reperfusion affects outcome in patients with benefit from EVT but not in patients without treatment benefit, we systematically assessed the importance of reperfusion level on functional outcome prediction using machine learning in patients with LVO stroke treated with EVT in clinical practice (*N* = 5235, German-Stroke-Registry) and in patients treated with EVT or best medical management from RCTs (*N* = 1488, Virtual-International-Stroke-Trials-Archive). The importance of reperfusion level on outcome prediction in an RCT-like real-world cohort equaled the importance of EVT treatment allocation for outcome prediction in RCT data and was higher compared to an unselected real-world population. The importance of reperfusion level was magnified in patient groups underrepresented in RCTs, including patients with lower NIHSS scores (0–10), M2 occlusions, and lower ASPECTS (0–5 and 6–8). Reperfusion level was equally important in patients with vertebrobasilar as with anterior LVO stroke. The importance of reperfusion level for outcome prediction identifies patient target groups who likely benefit from EVT, including vertebrobasilar stroke patients and among patients underrepresented in RCT patients with low NIHSS scores, low ASPECTS, and M2 occlusions.

**Supplementary Information:**

The online version contains supplementary material available at 10.1007/s12975-022-01040-5.

## Introduction

Multiple randomized controlled trials (RCTs) have provided high-level evidence for the efficacy of endovascular thrombectomy (EVT) in patients with acute ischemic stroke caused by large vessel occlusion (LVO) of the anterior circulation [1]. Accordingly, EVT treatment is strongly recommended for patients that comply with RCT inclusion and exclusion criteria [2, 3]. In contrast, the evidence level and class of recommendation are weaker for patients that were underrepresented in RCTs such as those with low symptom burden, medium vessel occlusions, and higher ischemic core volume on baseline imaging [3]. Similarly, whether EVT is beneficial for vertebrobasilar LVO stroke remains uncertain [4, 5]. Yet, these patient groups are considered for EVT in clinical practice, and knowledge whether EVT improves functional outcome in these patients would strongly aid clinical decision-making. Prospective registries depict real-world data including patient groups underrepresented in RCTs but that were treated in clinical practice. Providing information on the outcome of these patients, real-world data complement RCTs in providing evidence for treatments [6, 7, 8] — a potential that has recently been appraised by regulators [9, 10]. However, observational data from prospective registries usually lack a control group, are thus inherently challenging to utilize for the identification of patients that might benefit from treatment, and accordingly provide lower level of evidence and class of recommendation in guidelines.

To overcome this limitation, we here propose and employ a methodological framework to systematically analyze the effect of treatment on functional outcome in real-world data. We followed the premise that only in patients that benefit from EVT treatment the final reperfusion grade (successful vs. unsuccessful) would impact on outcome, whereas in patients without benefit from EVT, information on reperfusion would not impact on outcome. Accordingly, using the value of EVT treatment allocation for outcome prediction in RCT data as a reference, we assessed the value of the final reperfusion grade (final modified Thrombolysis in Cerebral Infarction (mTICI) score) for the prediction of functional outcome in real-world data. To account for dependencies among a larger number of non-linear baseline predictors as observed in stroke patients, we applied a machine-learning algorithm to determine the value of EVT treatment for the prediction of functional outcome in both real-world and RCT patients with LVO stroke. Recently, such variable importance analyses have increasingly been used to rank the predictive values of individual features [11, 12, 13] and to select patients for treatment [14, 15], but have never been applied in the context of patient selection for EVT. Here, we employed the importance of reperfusion level for outcome prediction as a marker to systematically evaluate which patients with anterior and vertebrobasilar stroke might benefit from EVT beyond those complying with RCT criteria.

## Methods

### Study Samples

#### German Stroke Registry — Endovascular Treatment

We retrieved data from 6635 patients from the German Stroke Registry — Endovascular Treatment (GSR, ClinicalTrials.gov Identifier: NCT03356392), an ongoing, academic, prospective, multicenter registry in Germany [16]. GSR inclusion criteria were a diagnosis of acute ischemic stroke due to LVO, initiation of EVT, and age > 18 years without any exclusion criteria. Between 2015 and 2019, patients were recruited in 25 centers distributed across Germany. We selected variables prior to arterial puncture (see Supplemental Methods for further details) as well as the final modified Thrombolysis in Cerebral Infarction (mTICI) scale score from the GSR database. We used the mTICI scale as a technical outcome measure of EVT indicating reperfusion level and grouped it into complete (3), substantial (2b), and no or minimal (0–2a) successful reperfusion. We excluded 1400 patients for lack of information and other reasons (see Supplemental Methods and Supplemental Fig. 1 for further details). The study was conducted in accordance with the Declaration of Helsinki and was approved both centrally by the Institutional Review Board (IRB) of the Ludwig-Maximilians-Universität Munich (protocol No 689–15) and by local IRBs.

#### Virtual International Stroke Trials Archive — Endovascular

We retrieved data from 1615 patients with ischemic stroke due to LVO in the anterior circulation treated with EVT or best medical care from completed RCTs from the Endovascular subsection of the Virtual International Stroke Trials Archive (VISTA-Endovascular, see Supplemental Methods and Supplemental Fig. 1 for database description and exclusion criteria) [17]. If available in the VISTA database, we retrieved the same variables from VISTA-Endovascular that were selected from the GSR database (see Supplemental Methods for exclusion criteria of variables).

Functional outcome was assessed using the mRS score ranging from 0 (no symptoms) to 6 (death) at 90 days. The primary outcome measure was functional independence (mRS 0–2) at 90 days.

### Machine Learning and Statistical Analyses

All analyses were performed in “R,” version 4.0.2.

#### Machine Learning Models

We predicted outcome with gradient boosting machines (GBMs). For each model respectively, data were randomly split into a training set (80%) and a test set (20%) with similar distribution of outcomes between sets. Within the training set, the model was trained and optimized with a repeated (*n* = 100) fivefold cross-validation. To avoid classification bias in a setting of uneven distribution of outcomes, we down-sampled data within each fold. For each model, we excluded patients with missing data (Supplemental Fig. 1). Predictive performances of machine-learning algorithms were determined in the test set and quantified as the AUC of the receiver operating characteristic (ROC). 95% confidence intervals of ROC curves were calculated using bootstrap replicates. ROC curves were compared with the Delong method. The value of each variable for the prediction of functional outcome was determined in the full datasets by assessing the change in model performance after permuting each variable a hundred times. A larger drop in performance corresponds to higher variable importance. To differentiate between analytical “importance” derived from these analyses and “importance” that interprets the strength of an effect or meaning, we have from here on indicated analytical *importance* by italic writing.

## Results

### Added Value of Reperfusion Level for Outcome Prediction

**Fig. 1:**
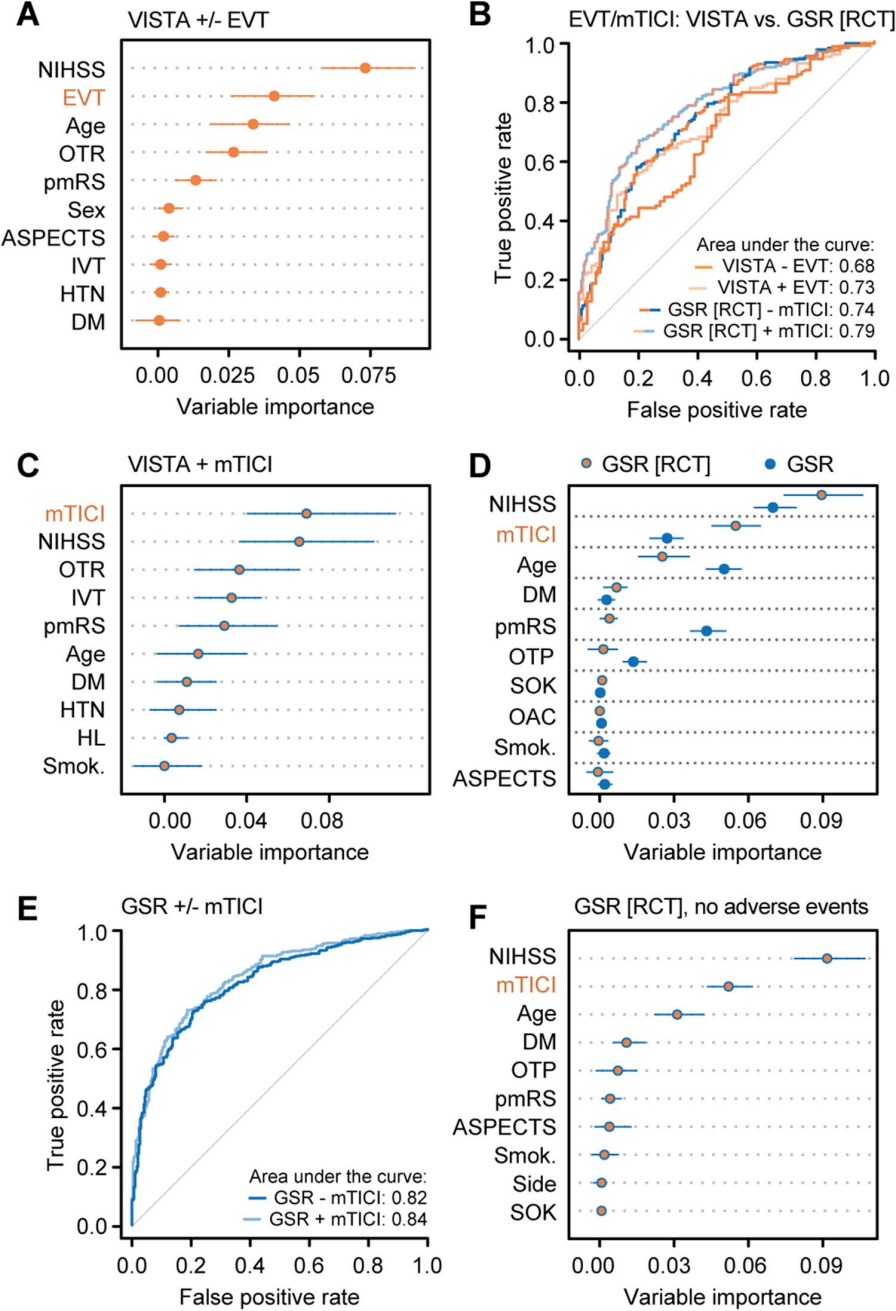
Added value of reperfusion level for outcome prediction in RCT and real-world data. **A** Allocation to EVT treatment was the second most important variable for outcome prediction in RCT data (VISTA, EVT and best medical care group). **B** Adding information on whether patients underwent EVT improved the pre-arterial puncture model for outcome prediction in RCT data (VISTA, EVT and best medical care group) as did adding the mTICI score in an RCT-like real-world cohort (GSR). **C** The mTICI score was the most important predictor in RCT data (EVT group only). **D** Selecting real-world patients that comply with RCT inclusion criteria increased the importance value of the mTICI score for outcome prediction compared to an unselected real-world cohort (GSR). **E** Adding the mTICI score to pre-arterial puncture variables in an unselected real-world dataset did not considerably improve outcome prediction. **F** mTICI score importance was similar in RCT-like real-world patients without adverse events during EVT. **A**, **C**, **D**, **F** Conditional variable importance analyses. Shown are the median and 5% and 95% quantiles the of importance values. ML, machine learning; GSR, German Stroke Registry; VISTA, Virtual International Stroke Trials Archive; HTN, hypertension; DM, diabetes mellitus; pmRS, premorbid modified Rankin Scale; GBM, gradient boosting machine; AUC, area under the curve; VB, vertebrobasilar; NIHSS, National Institutes of Health Stroke Scale; EVT, endovascular treatment; Smok., history of smoking; OAC, oral anticoagulation; SOK, known symptom onset; HL, hyperlipidemia; ASPECTS, Alberta Stroke Program Early CT Score; IVT, intravenous thrombolysis; mTICI, modified Thrombolysis in Cerebral Infarction; RCT, randomized controlled trial; OTP, onset-to-puncture time; OTR, onset-to-randomization time

To verify our approach of utilizing the value of a technical outcome measure (final mTICI score) for the prediction of functional outcome as a marker indicating whether patients would benefit from EVT, we first determined the value of EVT treatment allocation for outcome prediction in data from 1488 patients with anterior LVO stroke treated with EVT or best medical care from EVT RCTs (VISTA-Endovascular, Supplemental Fig. 1, Table 1). Information on whether patients underwent EVT was the second most *important* variable after the NIHSS score for outcome prediction in RCT data (Fig. 1A). Also, adding information on EVT treatment allocation to a classifier based on pre-arterial puncture parameters significantly improved outcome prediction in RCT data (AUC 0.73 [95% CI, 0.66–0.80] vs 0.68 [95% CI, 0.60–0.76], *p* = 0.007, Fig. 1B, Supplemental Fig. 2). In RCT patients treated with EVT, the mTICI score was the most *important* variable for outcome prediction (Fig. 1C) and numerically similar to EVT treatment allocation (Fig. 1A). Using these findings as a reference, we next hypothesized that the mTICI score would contribute more to outcome prediction in an RCT-like real-world cohort compared to unselected real-world cohort: we thus determined how the mTICI score contributes to outcome prediction in real-world patients with anterior LVO stroke (data from the German Stroke Registry – Endovascular Treatment [GSR], Table 1) complying with RCT criteria (age 18–80 years, time from symptom onset to arterial puncture < 12 h, pmRS score zero or one) [1]. In this cohort, the mTICI score ranked second among outcome predictors (Fig. 1D), numerically comparable to EVT treatment allocation as well as mTICI score *importance* in RCT data (Fig. 1A, C), and significantly improved a pre-arterial puncture classifier for outcome prediction (AUC 0.79 [95% CI, 0.75–0.83] vs 0.74 [95% CI, 0.70–0.79], *p* = 0.001, Fig. 1B, Supplemental Fig. 2). In contrast, the variable *importance* of the mTICI score for outcome prediction was considerably lower in an unselected real-world cohort (Fig. 1D) as was the numerical improvement of the pre-arterial puncture classifier by adding the mTICI score (AUC 0.84 [95% CI, 0.81–0.87] vs 0.82 [95% CI, 0.79–0.85], *p* = 0.006: Fig. 1E). Similar findings were obtained when applying a generalized linear model (Supplemental Fig. 3A). To evaluate whether the value of the graded treatment marker mTICI for outcome prediction would be explained by adverse events from EVT or rather the benefit associated with treatment, we performed a sensitivity analysis by excluding patients with adverse events during EVT and importantly found that the mTICI score *importance* for outcome prediction did not change when excluding these patients (Fig. 1F). Of note, we further found that pre-arterial puncture variables did not have any informative value for predicting the mTICI score (Supplemental Fig. 4).

**Table 1:** Baseline characteristics and procedural results of real-world and RCT patients

Characteristics	GSR-anterior *N* = 4666	GSR-VB *N* = 569	VISTA *N* = 717
Age, median (IQR) (years)	76 (66–83)	75 (65–82)	68 (57–76)
Female, no. (%)	2414 (51.7)	244 (43.0)	325 (45.3)
Medical history, no. (%)			
Hypertension	3585 (77.6)	450 (80.5)	380 (53.2)
Diabetes mellitus	1032 (22.3)	116 (21.1)	95 (13.3)
Dyslipidemia	1876 (40.6)	215 (39.1)	228 (32.8)
Atrial fibrillation	1987 (43.1)	188 (33.8)	–
Current smoking	653 (17.1)	74 (13.0)	194 (28.4)
Drug use, no. (%)			
Oral anticoagulation	982 (21.5)	88 (16.2)	–
Antiplatelet agents	1429 (31.3)	202 (37.2)	–
Pre-stroke mRS score, median (IQR)	0 (0–1)	0 (0–1)	0 (0–0)
Baseline NIHSS score, median (IQR)	15 (10–18)	16 (7–26)	17 (14–20)
Left side, no. (%)	2414 (51.9)	NA	–
Affected vessel, no. (%)			
M1	2818 (60.4)	NA	–
M2	1085 (23.3)	NA	–
ICA	564 (12.1)	NA	–
ICA-T	842 (18.0)	NA	–
Basilar artery	NA	532 (93.5)	–
Vertebral artery only	NA	37 (6.5)	–
General anesthesia, no. (%)	2946 (65.2)	493 (89.2)	–
Ship to interventional center, no. (%)	1970 (42.2)	235 (41.3)	–
Symptom onset known, no. (%)	2830 (60.7)	345 (60.6)	–
Out of hour admission, no. (%)	2772 (59.4)	324 (56.9)	–
High volume center, no. (%)	2873 (61.6)	367 (64.5)	–
ASPECTS, no. (%)			
High (9–10)	2205 (52.6)	NA	349 (48.7)
Middle (6–8)	1593 (38.0)	NA	301 (42.0)
Low (0–5)	377 (9.0)	NA	67 (9.3)
Intravenous alteplase treatment, no. (%)	2414 (52.0)	258 (45.6)	642 (89.5)
Symptom onset to arterial puncture, median (IQR) (min)	220 (153–334)	252 (164–399)	181 (142–237)
mTICI, no. (%)			
3	2282 (49.6)	344 (62.3)	–
2b	1609 (34.9)	133 (24.1)	–
0–2a	714 (15.5)	75 (13.6)	–

*mRS* modified Rankin Scale, *IQR* interquartile range, *NIHSS* National Institutes of Health Stroke Scale, *M1/2* first/second segment of the middle cerebral artery, *ICA* internal carotid artery, *ICA-T* internal carotid artery T, *ASPECTS* Alberta Stroke Program Early CT Score, *min* minutes, *mTICI* modified Thrombolysis in Cerebral Infarction, *VB* vertebrobasilar

### Added Value of Reperfusion Level in Patient Subgroups from Real-World Data

**Fig. 2:**
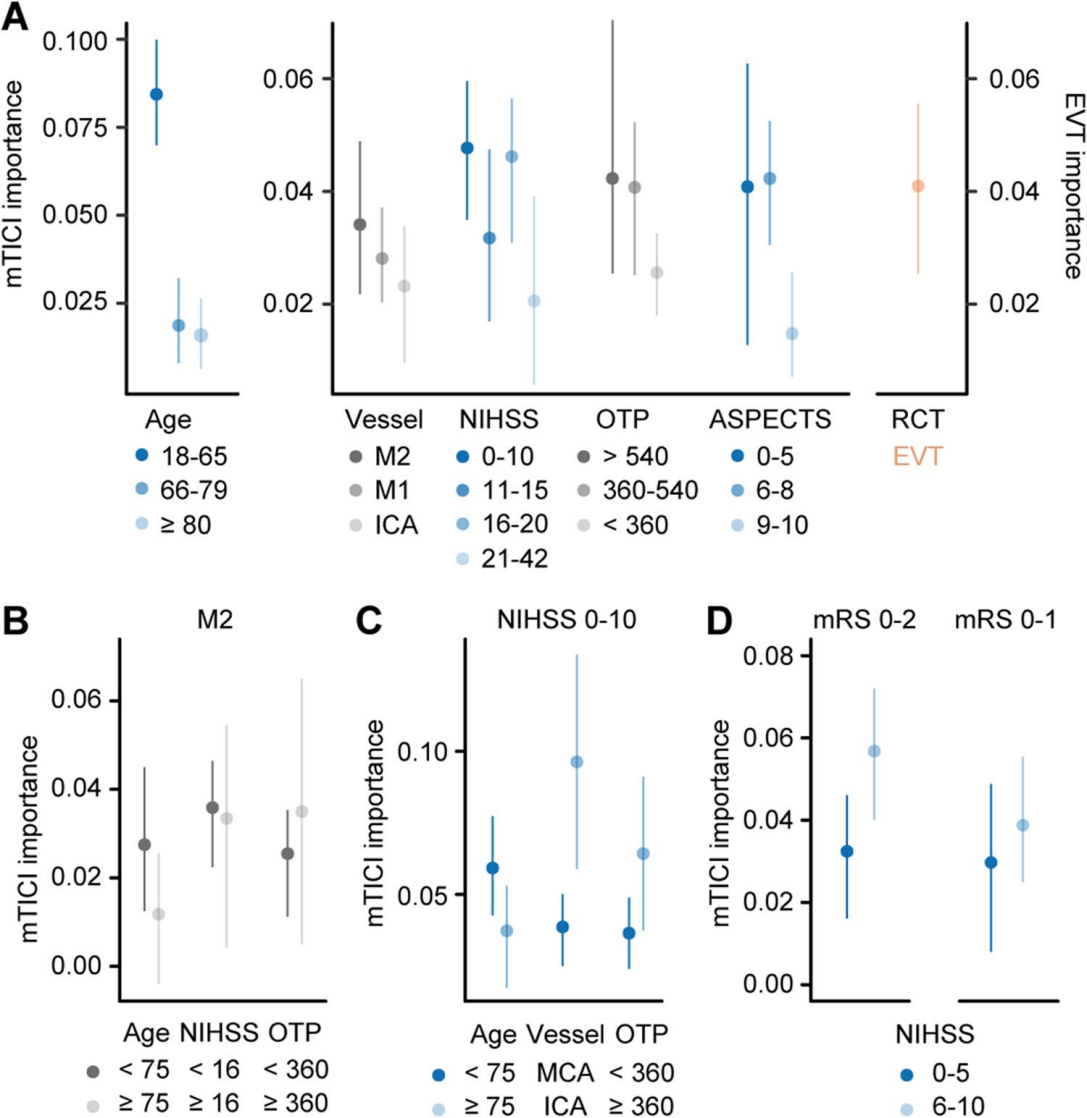
mTICI importance for outcome prediction across baseline strata in real-world data. **A** mTICI importance values for outcome prediction were higher in younger patients, in M2 occlusions, with lower NIHSS scores, longer onset-to-puncture times, and lower ASPECTS when compared to other subgroups of the respective strata and similar to the variable importance of EVT treatment allocation in RCT data. **B** In patients with M2 occlusions, mTICI importance was higher in younger patients. **C** In patients with lower NIHSS scores, mTICI importance was higher in ICA over MCA occlusions and with longer onset-to-puncture times. **D** Patients with NIHSS scores between 0 and 5 showed lower mTICI importance for outcome prediction compared to patients with NIHSS scores between 6 and 10 with a similar trend observed for the prediction of excellent outcome (mRS 0–1). **A**–**D** Conditional variable importance analyses in real-world data (GSR). Shown are the median and 5% and 95% quantiles of importance values. mTICI, modified Thrombolysis in Cerebral Infarction; NIHSS, National Institutes of Health Stroke Scale; OTP, onset-to-puncture time; ASPECTS, Alberta Stroke Program Early CT Score; M1, first segment of the middle cerebral artery; ICA, internal carotid artery; EVT, endovascular thrombectomy; RCT, randomized controlled trial; mRS, modified Rankin Scale

To identify patients that might benefit from EVT in real world beyond those complying with RCT criteria, we next determined the mTICI score variable *importance* for outcome prediction across subgroups of age, vessel location, the NIHSS score upon admission, onset-to-arterial puncture time, and the ASPECTS all stratified following approaches from a previous meta-analysis [1] and guidelines [3]. The mTICI score variable *importance* was higher in patients younger than 65 years compared to older patients, with lower (0–10) compared to higher (21–42) NIHSS scores, with longer (> 360 min) compared to shorter onset-to-arterial puncture times, and in those with lower (0–5) and medium (6–8) compared to higher (9–10) ASPECTS. Similar findings were obtained when applying a generalized linear model (Supplemental Fig. 3B). We further observed a trend for higher mTICI score variable *importance* in M2 compared to M1 and ICA occlusions. Values for patients with M2 occlusions, lower NIHSS, longer onset-to-puncture times, and lower APSECTS were similar to the value of EVT treatment allocation in RCT data (Fig. 2A). The distribution of the mTICI score was similar across subgroups indicating limited influence on its variable *importance* for outcome prediction (Supplemental Fig. 5). Considering the lower level of evidence for EVT efficacy in patients with M2 occlusions [18] and lower NIHSS scores [19], we next aimed to identify patients from these subgroups that might show a benefit from effective recanalization by EVT. Younger patients with M2 occlusions showed higher mTICI *importance* for outcome prediction than older patients, while there was no difference in mTICI *importance* between patients with higher and lower NIHSS (Fig. 2B). Among patients with lower NIHSS scores, those with lower age, ICA compared to MCA occlusions, and longer onset-to-puncture times showed higher mTICI *importance* for outcome prediction (Fig. 2C). The mTICI *importance* for outcome prediction was lower in patients with NIHSS scores between 0 and 5 compared to patients with NIHSS scores between 6 and 10 (Fig. 2D), but comparable to patients with NIHSS scores between 11 and 15 (Fig. 2A), with a similar trend for the prediction of excellent outcome (Fig. 2D).

### Value of Reperfusion Level for Outcome Prediction in Vertebrobasilar Stroke

**Fig. 3:**
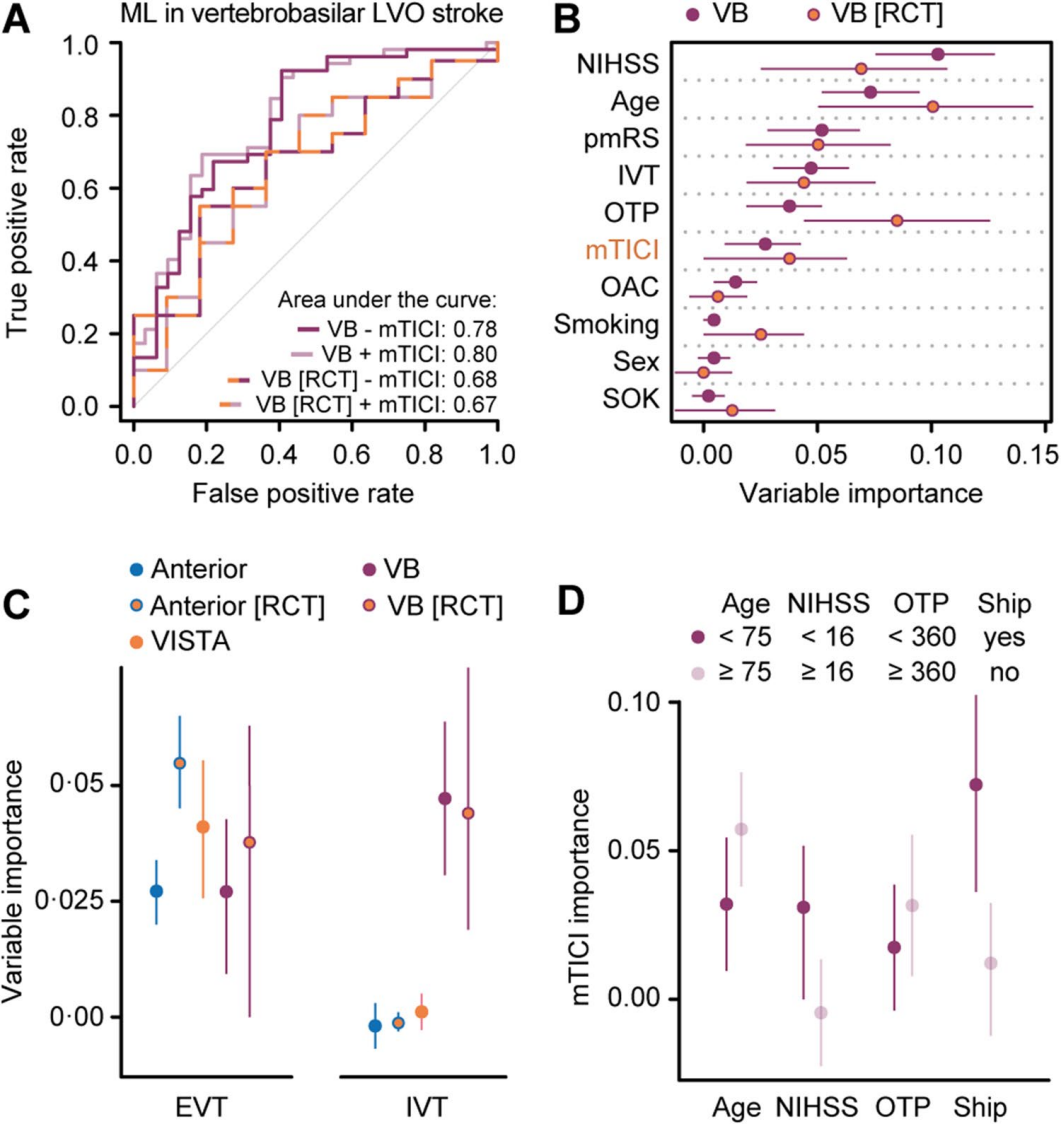
IVT treatment is more important than the mTICI score following EVT in vertebrobasilar LVO stroke. **A** Adding the mTICI score to pre-arterial puncture variables did not significantly improve the ML model for outcome prediction in both unselected vertebrobasilar LVO stroke patients and a selected cohort of patients complying with RCT inclusion criteria (basilar artery occlusion, NIHSS score > 9, age 18 to 85 years, onset-to-puncture time < 6 h, pmRS score 0 to 2). **B** Selecting real-world patients that comply with RCT inclusion criteria slightly increased the importance value of the mTICI score for outcome prediction. **C** While the importance values of the mTICI score for outcome prediction were similar between anterior and vertebrobasilar datasets, the IVT treatment importance value was consistently higher in vertebrobasilar compared to anterior LVO stroke. **D** The mTICI score importance value was higher in older patients, with lower NIHSS scores, and in patients undergoing interhospital transfer. **B**–**D** Conditional variable importance analyses in real-world data (GSR) and RCT data (VISTA, **C**). Shown are the median and 5% and 95% quantiles of importance values. ML, machine learning; VB, vertebrobasilar; LVO, large-vessel occlusion; RCT, randomized controlled trial; mTICI, modified Thrombolysis in Cerebral Infarction; NIHSS, National Institutes of Health Stroke Scale; OTP, onset-to-puncture time; pmRS, premorbid modified Rankin Scale; IVT, intravenous thrombolysis; OAC, oral anticoagulation; SOK, known symptom onset; EVT, endovascular treatment; ship, interhospital transfer

The added value of EVT in vertebrobasilar LVO stroke remains uncertain [20]. Here, using real-world data from 569 patients that underwent EVT following vertebrobasilar LVO stroke (Table 1), we found that adding the mTICI score to pre-arterial puncture variables did not significantly improve the pre-arterial puncture classifier (AUC 0.80 [95% CI, 0.70–0.90] vs. 0.78 [95% CI, 0.68–0.89], *p* = 0.18), also not when selecting real-world patients that comply with BASICS inclusion criteria (age 18–85 years, time from symptom onset to arterial puncture < 6 h, pmRS score < 3, NIHSS score > 9; AUC 0.67 [95% CI, 0.46–0.88] vs 0.68 [95% CI, 0.48–0.88], *p* = 0.89: Fig. 3A, Supplemental Figs. 1, 2) [21]. In real-world data, the variable *importance* of the mTICI score for outcome prediction ranked only sixth and was lower than the NIHSS score upon admission, age, the pmRS score, treatment with intravenous thrombolysis (IVT), and the onset-to-puncture time. Its value slightly increased in patients complying with BASICS inclusion criteria (Fig. 3B). While the variable *importance* of reperfusion by EVT was similar between anterior and vertebrobasilar LVO stroke with patient selection based on RCT inclusion criteria generally leading to higher values, the variable *importance* of additional IVT treatment was consistently higher in vertebrobasilar compared to anterior LVO stroke (Fig. 3C). To identify subgroups of patients with vertebrobasilar LVO stroke that might benefit from EVT, we determined mTICI score variable *importance* across different baseline strata and found a greater contribution to outcome prediction in patients older than 75 years, with lower NIHSS scores, and in patients shipped from primary to interventional centers (Fig. 3D).

## Discussion

To identify LVO stroke patients that might benefit from EVT but were underrepresented in RCTs and following the premise that only in patients with benefit from EVT successful reperfusion would contribute to outcome prediction, we systematically assessed the value of the level of reperfusion for functional outcome prediction using a machine learning algorithm in data from > 6700 patients from both a large prospective multicenter real-world registry and from RCTs on EVT efficacy. We found that reperfusion level (assessed by the final mTICI score) was of similar *importance* for the prediction of functional outcome in RCT and RCT-like real-world patients as EVT allocation in RCT patients, which ranked second for outcome prediction in RCT data, in line with the high-level evidence for EVT from RCTs. The contribution of reperfusion level to outcome prediction was higher in an RCT-like compared to an unselected real-world cohort, thus correctly detecting higher EVT efficacy in the RCT-like cohort and endorsing its value as a marker for EVT treatment benefit. We thus utilized the value of reperfusion level for outcome prediction as a marker for treatment benefit in real-world data and identified patients that were largely excluded from RCTs but might benefit from EVT: patients with a lower NIHSS score, M2 occlusions, longer onset-to-puncture times, and a lower ASPECTS as well as older patients with vertebrobasilar LVO. Demonstrating the value of a technical outcome measure for the prediction of functional outcome, our study has the potential to inform future clinical trial design and influence clinical decisions.

While RCTs are indispensable to demonstrate treatment efficacy, they are usually restricted to selected patient populations. In contrast, real-world registry data are more comprehensive and usually include significantly more patients but are observational by nature and lack an untreated control group to allow investigation of treatment efficacy in subgroups excluded from or underrepresented in RCTs. Using a combinatory approach, we here drew on the advantages of both RCTs and real-world registry data to establish and apply the value of reperfusion level for outcome prediction as a marker that indicates benefit from EVT. Our data are in line with studies reporting reperfusion level as a strong predictor of outcome [22, 23]. However, in contrast to the well-known evidence for the link between the degree of reperfusion and functional outcome, we here determined in which patients successful reperfusion impacts functional outcome the most, by applying variable *importance* analyses. Specifically, in patients with benefit from EVT treatment, variable *importance* of mTICI is high, whereas in patients without substantial benefit from EVT treatment, information on recanalization (mTICI 2b/3 vs. 0) does not impact functional outcome considerably. Determining variable *importance* using machine learning has gained increasing attention across medical fields [11, 12, 15], but has never been applied in the context of our study goal. Here, variable *importance* analyses allowed us to assess the value of reperfusion level in conjunction with all available clinical variables, which is in contrast to the prevailing strategy to adjust only for pre-selected variables. To further consolidate whether the value of reperfusion level for functional outcome prediction could be interpreted as treatment benefit from EVT, future studies might also apply traditional statistical approaches such as logistic regression to compare odds ratios for the association of the level of reperfusion with functional outcome between different patient subgroups.

Following the established evidence for EVT efficacy in patients with anterior LVO stroke [1], there has been high interest to identify patient subgroups excluded from or underrepresented in these RCTs that would also benefit from EVT. Here, we found similar variable *importance* of reperfusion level for outcome prediction in patients with longer onset-to-puncture times and in patients with lower ASPECTS compared to EVT variable *importance* in RCT patients. This is in line with RCTs demonstrating EVT efficacy for extended time windows [24, 25] and encourages ongoing trials for lower ASPECTS, respectively [26]. While lower ASPECTS is associated with worse outcome when analyzed cross-sectionally [27], this does not necessarily imply that the treatment effect for this subgroup is smaller than for patients with higher ASPECTS: compared to patients from the pivotal RCT trials forming the HERMES study group with a median ASPECTS of 9 for which EVT increased the likelihood for good outcome at 90 days by 19.5% [1], patients with a large ischemic core (ASPECTS 3–5) from a recent Japanese RCT showed an EVT treatment benefit of 18.3% risk difference for the mRS score 0–3 at 90 days [28]. We also found similar variable *importance* of the mTICI score in patients with lower NIHSS scores in accordance with a recent meta-analysis indicating similar treatment effects of EVT in this subgroup [19]. Among patients with lower NIHSS scores upon admission, we found higher variable *importance* of the mTICI score for outcome prediction in younger patients, ICA rather than MCA occlusion, and longer onset-to-puncture times pointing towards treatment benefits in these subgroups. Collectively, these findings might describe a scenario, in which younger patients with mild symptoms show progressive tissue loss if not treated with EVT as their collateral flow, initially sufficient to perfuse most of the affected tissue, breaks down over time. Lastly, we observed a trend for higher *importance* of reperfusion level for outcome prediction in patients with M2 occlusions compared to M1 occlusions, substantiating the demand for an RCT investigating EVT efficacy in patients with medium vessel occlusion [18]. Such a trial could particularly recruit patients < 75 years, which showed high value of reperfusion level for outcome prediction. It is important to note that we observed overlapping confidence intervals for the importance of the mTICI score for outcome prediction between most subgroups, indicating that more work on other datasets is necessary to validate our findings.

In contrast to anterior circulation LVO stroke, the evidence for EVT efficacy in vertebrobasilar LVO stroke remains uncertain [4, 5, 20]. Analyzing a real-world cohort of 569 patients, we found similar variable *importance* of reperfusion level for outcome prediction in patients with anterior and vertebrobasilar LVO stroke. Focusing on subgroups of vertebrobasilar LVO stroke patients, we found longer onset-to-puncture times to be linked to higher variable *importance* of reperfusion level for outcome prediction, which is in line with data from the BASILAR registry [4], which allowed recruitment up to 24 h and showed higher EVT effects than the BASICS study [5]. We also observed that older patients with vertebrobasilar LVO stroke showed high variable *importance* of reperfusion level for outcome prediction in agreement with a subgroup analysis from the BASICS study [5]. Furthermore, IVT treatment showed a remarkably high variable *importance* for outcome prediction in vertebrobasilar LVO stroke patients undergoing EVT in line with the BASICS study having the highest IVT rate but lowest absolute risk reduction for poor outcome by EVT compared to the BEST study [20] and the BASILAR registry.

Our study has several strengths. We leveraged data from both a large real-world registry and from several RCTs on EVT efficacy covering > 6700 patients. Our sample size exceeded the sample size of previous studies on machine learning for the prediction of outcome after stroke by a factor of 3–10 [29–31] and allowed to establish a machine learning classifier that was superior to other machine learning models [29, 30, 31] and summative scores ^32,33^. In contrast, while allowing for the most important subgroup analyses, our sample size was insufficient to further subdivide subgroups such as patients with lower ASPECTS or to study patients with exclusive occlusions of the posterior or anterior cerebral artery. Our study is also limited by a potential selection bias in real-world data that could have influenced variable *importance* values for outcome prediction, for example by selecting only patients with dominant M2 (over non-dominant) occlusions for EVT and not selecting severely affected patients with ICA occlusion. It is further limited by the potential personal bias on mTICI evaluation and its limited validation for occlusion of the distal anterior or vertebrobasilar circulation. Lastly, a higher predictive treatment value might have also resulted if the treatment was associated with significant adverse events that impact on outcome. Here, we showed that the mTICI score value for outcome prediction is independent of adverse events occurring during EVT indicating that it must rather be the relation of higher mTICI scores with better outcome that drives the value of the mTICI score for outcome prediction.

In conclusion, we here identified several subgroups not well represented in RCTs that might also benefit from EVT including patients with a lower NIHSS score, M2 occlusions, longer onset-to-puncture times, and lower ASPECTS. Our data suggest that IVT treatment has high variable *importance* for outcome prediction in patients with vertebrobasilar LVO stroke. Our ML-based approach could serve as a blueprint for other medical fields on how to utilize the value of a graded treatment marker for outcome prediction in real-world and RCT data to inform clinical decisions and future RCT design.

## Supplementary Information

Below is the link to the electronic supplementary material.

Supplementary file1 (DOCX 22 KB)

Supplementary file2 (PDF 75 KB)

Supplementary file3 (PDF 63 KB)

Supplementary file4 (PDF 80 KB)

Supplementary file5 (PDF 89 KB)

Supplementary file6 (PDF 64 KB)

## Data Availability

The data that support the findings of this study were obtained from the German Stroke Registry (GSR) and the Virtual International Stroke Trials Archive (VISTA) but restrictions apply to the availability of these data, which were used under license for the current study, and so are not publicly available. The R-script used for the current study can be obtained from the first author upon reasonable request.
