# Supplementary material for: Machine Learning–Based Identification of Target Groups for Thrombectomy in Acute Stroke
Source: Transl Stroke Res. 2022 Jun 7;14(3):311–21. doi: 10.1007/s12975-022-01040-5 (PMC10159968; doi:10.1007/s12975-022-01040-5)
Supplement: Supplementary file 1 — Supplementary file1 (DOCX 22 KB) [file 12975_2022_1040_MOESM1_ESM.docx]

**SUPPLEMENTAL MATERIAL**

**Machine learning-based identification of target groups for thrombectomy in acute stroke**

Fanny Quandt, MD, Fabian Flottmann, MD, Vince I Madai, MD, PhD, Anna Alegiani, MD, Clemens Küpper, MD, Lars Kellert, MD, Adam Hilbert, Dietmar Frey, MD, Thomas Liebig, MD, Jens Fiehler, MD, Mayank Goyal, MD, Jeffrey L Saver, MD, Christian Gerloff, MD, Götz Thomalla, MD, Steffen Tiedt, MD, PhD, on behalf of the GSR investigators and the VISTA-Endovascular Collaborators.

**List of content:**

Supplemental Methods

Supplemental Tables I and II

Supplemental Figures I – V

**Supplemental Methods. Predictor variable selection and data preprocessing**

**German Stroke Registry – Endovascular Treatment**

Out of data from 6,635 patients from the German Stroke Registry – Endovascular Treatment, we excluded 1,400 patients that lacked information on outcome or site of vessel occlusion, had incomplete EVT, exclusive occlusions of the anterior or posterior cerebral artery, or had an onset-to-puncture time > 36 hours (Supplemental Figure 1). We only included variables with less than 5 % missing values and, owing to its clinical importance, the time from symptom onset to arterial puncture and ASPECTS (10 % missing values each) resulting in 25 variables (Table). Information on antiplatelet and oral anticoagulation medication was binarized for both classes to yes/no. Admission time was grouped for out of office hour status defined as admission at weekends or between 4 pm and

8 am. Location of LVO was grouped into the first segment of the middle cerebral artery (M1), second segment of the middle cerebral artery (M2), intracranial internal carotid artery with and without T (ICA-T and ICA), and vertebrobasilar circulation. ASPECTS was grouped into not available, low (0-5), middle (6-8), and high (9-10). Centers were grouped according to their EVT volume dichotomized at 80 treatments per year. If symptom onset was unknown, we calculated the mean time between last seen well and time of recognition.

**Virtual International Stroke Trials Archive – Endovascular**

VISTA-Endovascular encompasses data from RCTs that meet the following criteria: a minimum dataset of 20 participants, baseline assessment conducted within 24 hours of stroke onset including information on neurological impairment and any type of vessel imaging, and functional outcome assessed using the modified Rankin Scale (mRS) score at 90 days after stroke onset. We excluded 127 patients with missing information on outcome or the Alberta Stroke Program Early CT Score (ASPECTS). We retrieved the same variables from VISTA-Endovascular that were selected from the GSR database. Also, we only included variables with less than 5 % missing values and, owing to its clinical relevance, the pre-stroke modified Rankin Scale (pmRS) score (31 % missing values) resulting in eleven variables (Table). As time from symptom onset to arterial puncture was not available in a sufficiently large number of patients (41 % missing values), we used the time from symptom onset to randomization as a surrogate.

**R packages**

Model prediction was performed using the ‘R’ package ‘caret’ version 6.0.86. ROC curves were calculated and compared using ‘R’ package ‘pROC’ version 1.16.2. Variable importance analyses was performed using the ‘R’ package ‘iml’ version 0.10.0).

**Supplemental Table I: List of GSR investigators**

| **GSR Investigators** | **GSR-ET Center** |
| --- | --- |
| J Berrouschot, A Bormann | Department of Neurology and Department of Neuroradiology, Klinikum Altenburger Land, Germany |
| G Bohner, CH Nolte, E Siebert, S Zweynert | Department of Neuroradiology, Institute of Neuroradiology and Department of Neurology, Charite Universitary Medicine Berlin, Germany |
| F Dorn, GC Petzold | Department of Neurology and Department of Neuroradiology, University Hospital Bonn, Germany / German Center for Neurodegenerative Diseases, Bonn, Germany |
| F Keil, W Pfeilschifter | Department of Neurology and Department of Neuroradiology, Institute of Diagnostic and Interventional Neuroradiology, University Hospital Frankfurt, Frankfurt am Main, Germany |
| GF Hamann | Department of Neurology, Bezirkskrankenhaus Günzburg, Germany |
| M Braun | Department of Radiology, Sektion Neuroradiologie Klinik für Diagnostische und Interventionelle Radiologie, Universitätsklinikum Ulm, BKH-Günzburg, Germany |
| B Eckert, J Röther | Department of Neuroradiology, Institut für Radiologie und Neuroradiologie and Department of Neurology, Neurologische Abteilung, Asklepios Klinik Altona, Hamburg, Germany |
| A Alegiani, J Fiehler, C Gerloff, G Thomalla | Department of Neurology and Department of Neuroradiology, University Medical Center Hamburg-Eppendorf, Germany |
| C Kraemer | Department of Neurology, Städtisches Klinikum Lüneburg, Germany |
| K Gröschel, T Uphaus | Department of Neurology, University Medical Center of the Johannes Gutenberg University Mainz, Mainz, Germany |
| L Kellert, S Tiedt, C Trumm | Department of Neurology, Institute of Neuroradiology, Institute for Stroke and Dementia Researh, University Hospital, LMU Munich, Munich, Germany |
| T Boeckh-Behrens, S Wunderlich | Department of Neuroradiology and Department of Neurology, Clinic and Policlinic for Neurology, Klinikum rechts der Isar, Technical University Munich, Germany |
| KH Henn, A Ludolph | Department of Neurology, Sana Klinikum Offenbach, Germany |
| M Petersen, F Stögbauer | Department of Neurology and Department of Radiology, Klinikum Osnabrück, Germany |
| U Ernemann, S Poli | Department of Neurology With Focus on Neurovascular Diseases, University Hospital Tübingen, Tübingen, Germany |

**Supplemental Table II: List of VISTA-Endovascular Collaboratos**

| **VISTA-Endovascular Investigators** | **Affiliation** |
| --- | --- |
| P Khatri (Chair) | Department of Neurology and Rehabilitation Sciences, University of Cincinnati, Cincinnati, OH, USA |
| M Bendszuz | Department of Neuroradiology, Heidelberg University Hospital, Heidelberg, Germany |
| S Bracard | Department of Diagnostic and Interventional Neuroradiology, INSERM U 947, Université de Lorraine and University Hospital of Nancy, France |
| J Broderick | University of Cincinnati Gardner Neuroscience Institute, OH, USA |
| B Campbell | Department of Medicine and Neurology, Melbourne Brain Centre at the Royal Melbourne Hospital, University of Melbourne, Parkville, Australia |
| A Ciccone | Department of Neurology and Stroke Unit, Carlo Poma Hospital, ASST Mantova, Mantua, Italy |
| A Davalos | Department of Neuroscience, Hospital Germans Trias i Pujol, Universitat Autònoma de Barcelona, Barcelona, Spain |
| S Davis | Department of Medicine and Neurology, Melbourne Brain Centre at the Royal Melbourne Hospital, University of Melbourne, Parkville, Australia |
| A Demchuk | Calgary Stroke Program, Departments of Clinical Neurosciences and Radiology, Hotchkiss Brain Institute, Cumming School of Medicine, University of Calgary, Canada |
| HC Diener | Department of Neurology, University Hospital Essen University Duisburg-Essen, Germany |
| D Dippel | Department of Neurology, Erasmus MC University Medical Center, Rotterdam, the Netherlands |
| GA Donnan | The Florey Institute of Neuroscience and Mental Health, University of Melbourne, Parkville, Australia |
| X Ducrocq | Neurology Department, Metz-Thionville Hospital, France |
| J Fiehler | Department of Diagnostic and Interventional Neuroradiology, University Medical Center Hamburg-Eppendorf, Hamburg, Germany |
| D Fiorella | Department of Neurosurgery, Stony Brook University, NY, USA |
| G Ford | Stroke Unit, Oxford University Hospitals and Division of Medical Sciences, Oxford University, United Kingdom |
| M Goyal | Calgary Stroke Program, Departments of Clinical Neurosciences and Radiology, Hotchkiss Brain Institute, Cumming School of Medicine, University of Calgary, Canada |
| W Hacke | Department of Neurology, University of Heidelberg, Heidelberg, Germany |
| M Hill | Calgary Stroke Program, Departments of Clinical Neurosciences, Medicine, Community Health Sciences, and Radiology, Hotchkiss Brain Institute, Cumming School of Medicine, University of Calgary, Canada |
| R Jahan | Division of Interventional Neuroradiology, Department of Radiological Sciences, University of California, Los Angeles (UCLA) |
| E Jauch | Mission Research Institute, Mission Health System, Asheville, NC, USA |
| T Jovin | Department of Neurology, Cooper University Hospital Neurological Institute, Camden, NJ, USA |
| C Kidwell | Department of Radiology, University of Arizona, Tucson, USA |
| KR Lees | Medical School and Institute of Cardiovascular and Medical Sciences, University of Glasgow, Glasgow, UK |
| DS Liebeskind | Department of Neurology, David Geffen School of Medicine at University of California Los Angeles |
| CB Majoie | Department of Radiology, Academic Medical Center Amsterdam, the Netherlands |
| S Martins | Department of Neurology, Federal University of Rio Grande do Sul and Hospital de Clínicas de Porto Alegre, Brazil |
| P Mitchell | Department of Radiology, Royal Melbourne Hospital, University of Melbourne, Parkville, Australia |
| J Mocco | Department of Neurosurgery, Icahn School of Medicine at Mount Sinai, New York City, NY, USA |
| K Muir | Institute of Neuroscience and Psychology, University of Glasgow, Scotland, United Kingdom |
| RG Nogueira | Department of Neurology, Emory University School of Medicine, Marcus Stroke & Neuroscience Center, Grady Memorial Hospital, Atlanta, GA, USA |
| JL Saver | Department of Neurology, David Geffen School of Medicine at the University of California, Los Angeles, Los Angeles, CA, USA |
| WJ Schonewille | Department of Neurology, St. Antonius Hospital, Nieuwegein, the Netherlands |
| AH Siddiqui | Department of Neurosurgery, State University of New York at Buffalo, USA |
| G Thomalla | Department of Neurology, University Medical Center Hamburg-Eppendorf, Hamburg, Germany |
| TA Tomsick | Department of Radiology, University of Cincinnati Academic Health Center, University Hospital, Cincinnati, Ohio, USA |
| AS Turk | Department of Radiology, Medical University of South Carolina, Charleston, USA |
| WH van Zwam | Department of Radiology, Maastricht University Medical Center Maastricht, the Netherlands |
| P White | Institute of Neuroscience, Newcastle University, Newcastle upon Tyne, UK |
| S Yoshimura | Department of Neurosurgery, Hyogo College of Medicine, Nishinomiya, Japan |
| OO Zaidat | Department of Neuroscience, St Vincent Mercy Hospital, Toledo, Ohio, USA |
