# Supplementary material for: Machine Learning–Based Identification of Target Groups for Thrombectomy in Acute Stroke
Source: Transl Stroke Res. 2022 Jun 7;14(3):311–21. doi: 10.1007/s12975-022-01040-5 (PMC10159968; doi:10.1007/s12975-022-01040-5)
Supplement: Supplementary file 2 — Supplementary file2 (PDF 75 KB) [file 12975_2022_1040_MOESM2_ESM.pdf]

Supplemental Figure 1

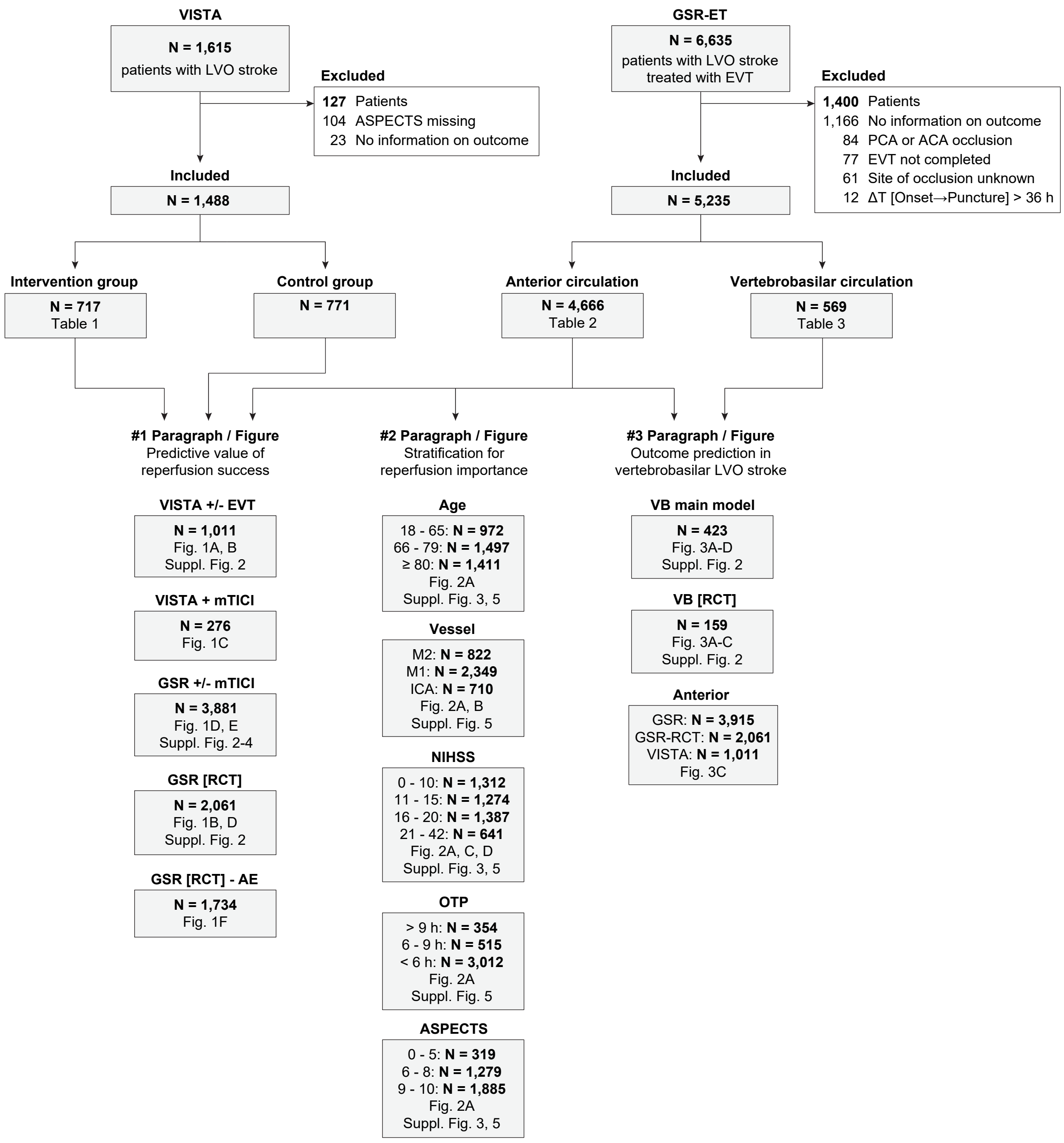

Flowchart of patients with LVO stroke included into analyses. Different patient numbers resulted from exclusion of patients with missing data for the respective analysis. GSR, German Stroke Registry; VISTA, Virtual International Stroke Trials Archive; LVO, large-vessel occlusion; EVT, endovascular treatment; PCA, posterior cerebral artery; ACA, anterior cerebral artery; mTICI, modified Thrombolysis in Cerebral Infarction; ΔT, time difference; ASPECTS, Alberta Stroke Program Early CT Score; Fig. figure; h, hour; M1/2, first/second segment of the middle cerebral artery; ICA, internal carotid artery; RCT, randomized controlled trial; OTP, onset-to-puncture time; mTICI, modified Thrombolysis in Cerebral Infarction; VB, vertebrobasilar; AE, adverse events.
