# Supplementary material for: Machine Learning–Based Identification of Target Groups for Thrombectomy in Acute Stroke
Source: Transl Stroke Res. 2022 Jun 7;14(3):311–21. doi: 10.1007/s12975-022-01040-5 (PMC10159968; doi:10.1007/s12975-022-01040-5)
Supplement: Supplementary file 3 — Supplementary file3 (PDF 63 KB) [file 12975_2022_1040_MOESM3_ESM.pdf]

Supplemental Figure 2

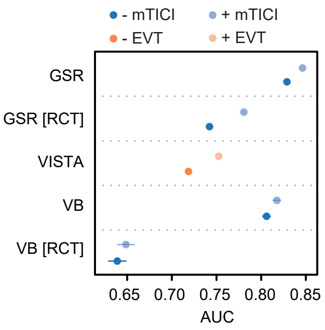

Model performance in training data. GBM performance of resampled training datasets for the different models. mRS, modified Rankin Scale; GBM, gradient boosting machine; AUC, area under the curve; GSR, german stroke registry; RCT, randomized controlled trial; VISTA, Virtual International Stroke Trials Archive; VB, vertebrobasilar; EVT, endovascular thrombectomy; mTICI, modified Thrombolysis in Cerebral Infarction scale.
