# Supplementary material for: Machine Learning–Based Identification of Target Groups for Thrombectomy in Acute Stroke
Source: Transl Stroke Res. 2022 Jun 7;14(3):311–21. doi: 10.1007/s12975-022-01040-5 (PMC10159968; doi:10.1007/s12975-022-01040-5)
Supplement: Supplementary file 4 — Supplementary file4 (PDF 80 KB) [file 12975_2022_1040_MOESM4_ESM.pdf]

Supplemental Figure 3

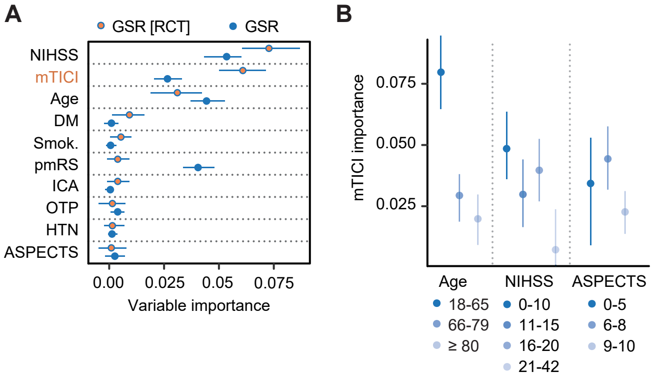

mTICI importance for outcome prediction using a generalized linear model. (**A**) Selecting real-world patients that comply with RCT inclusion criteria increased the importance value of the mTICI score for outcome prediction compared to an unselected real-world cohort (GSR). (**B**) mTICI importance values for outcome prediction were higher in younger patients, with lower NIHSS scores, and low-to-mid ASPECTS when compared to other subgroups of the respective strata. GSR, German Stroke Registry; RCT, randomized controlled trial; mTICI, modified Thrombolysis in Cerebral Infarction; pmRS, premorbid modified Rankin Scale; HTN, hypertension; ICA, internal carotid artery; NIHSS, National Institutes of Health Stroke Scale; ASPECTS, Alberta Stroke Program Early CT Score; DM, diabetes mellitus; Smok., smoking; OTP, onset to puncture time.
