# Supplementary material for: Machine Learning–Based Identification of Target Groups for Thrombectomy in Acute Stroke
Source: Transl Stroke Res. 2022 Jun 7;14(3):311–21. doi: 10.1007/s12975-022-01040-5 (PMC10159968; doi:10.1007/s12975-022-01040-5)
Supplement: Supplementary file 5 — Supplementary file5 (PDF 89 KB) [file 12975_2022_1040_MOESM5_ESM.pdf]

Supplemental Figure 4

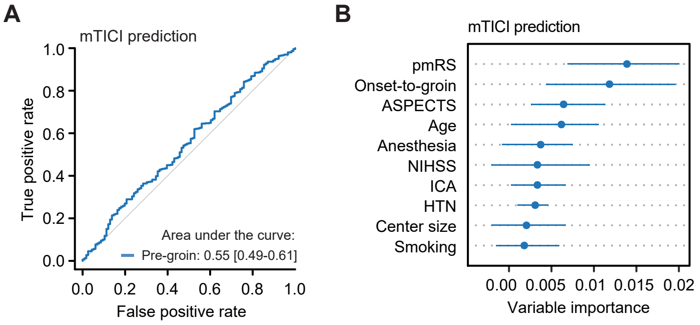

Pre-groin information does not predict reperfusion grade. Cumulative (A) and individual importance of pre-groin variables for predicting the final reperfusion grade mTICI (B) in the GSR. mTICI, modified Thrombolysis in Cerebral Infarction; pmRS, premorbid modified Rankin Scale; HTN, hypertension; ICA, internal carotid artery; IVT, intravenous thrombolysis; M2, second segment of the middle cerebral artery; NIHSS, National Institutes of Health Stroke Scale; ASPECTS, Alberta Stroke Program Early CT Score.
