# Supplementary material for: Machine Learning–Based Identification of Target Groups for Thrombectomy in Acute Stroke
Source: Transl Stroke Res. 2022 Jun 7;14(3):311–21. doi: 10.1007/s12975-022-01040-5 (PMC10159968; doi:10.1007/s12975-022-01040-5)
Supplement: Supplementary file 6 — Supplementary file6 (PDF 64 KB) [file 12975_2022_1040_MOESM6_ESM.pdf]

Supplemental Figure 5

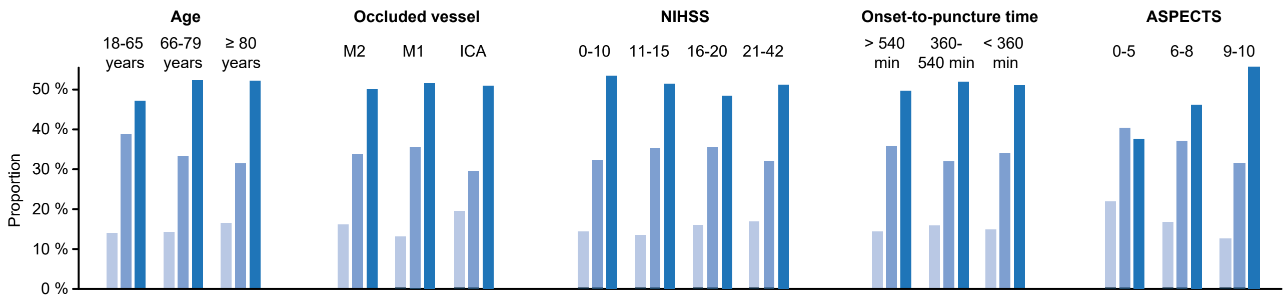

Similar distribution of mTICI scores across subgroups. The proportion of final mTICI scores was similar across subgroups of age, occluded vessel, the NIHSS score upon admission, the onset-to-puncture time, and the ASPECTS. The shades of the color blue indicate mTICI scores 0-2a (light blue), 2b (medium blue), and 3 (dark blue). mTICI, modified Thrombolysis in Cerebral Infarction; M1/2, first/second segment of the middle cerebral artery; NIHSS, National Institutes of Health Stroke Scale; ASPECTS, Alberta Stroke Program Early CT Score; ICA, internal carotid artery.
